# Crystal structure of the vicilin from *Solanum melongena* reveals existence of different anionic ligands in structurally similar pockets

**DOI:** 10.1038/srep23600

**Published:** 2016-03-23

**Authors:** Abha Jain, Ashish Kumar, Dinakar M. Salunke

**Affiliations:** 1 Regional Centre for Biotechnology, Faridabad-121001, India; 2 Manipal University, Manipal, Karnataka-576104, India; 3 National Institute of Immunology, New Delhi-110067, India; 4 International Centre for Genetic Engineering and Biotechnology, New Delhi-110067, India

## Abstract

Crystal structure of a vicilin, SM80.1, was determined towards exploring its possible physiological functions. The protein was purified from *Solanum melongena* by combination of ammonium sulphate fractionation and size exclusion chromatography. Structure was determined *ab initio* at resolution of 1.5 Å by X-ray crystallography showing the three-dimensional topology of the trimeric protein. Each monomer of SM80.1 consists of two similar domains with hydrophobic binding pocket and each accommodating different ligands, i.e. acetate and pyroglutamate. The relatively high stability of these independent anionic ligands in similar pockets indicated a strict requirement of stabilization by hydrogen bonds with the charged residues, suggesting a degree of plasticity within the binding pocket. Comparison of SM80.1 structure with those of other 7S vicilins indicated conservation of putative binding pocket for anionic ligands. Here we propose the possibility of trapping of these ligands in the protein for their requirement in the metabolic processes.

Seed proteins play a vital role in cellular growth and development, nutrient accumulation^1^, thiamine storage^2^, plant defense^3^, regulation of indole acetic acid (IAA) levels^4^. They have been shown to exhibit antimicrobial and antifungal activity^5^,^6^, hemagglutination activity^7^, desiccation tolerance^8^, ribosome inhibitory activity^9^ and many more. This suggests the seed proteins are indispensible for germination and further growth and development. Seed proteins are classified into four groups on the basis of solubility- albumin, globulin, prolamins and glutelins^10^.

Among the abundant seed proteins, globulins are considered a major family of seed proteome. Members of this family are classified on the basis of their sedimentation coefficient as 7S or 11S^11^. 7S globulins are also called vicilins and perform various functions, including the role in sucrose binding^12^, desiccation^13^, defense against microbes^14^ and oxidative stress^15^. The vicilin peptides formed by trypsin or chymotrypsin digestion exhibit antihypertensive effects^16^. Vicilins are considered to be the most potent class of allergenic proteins from seed proteome^17^.

Structurally, vicilins are trimeric, formed predominantly by non-covalent interactions. Canavalin, Phaseolin, β-conglycinin and AraH1 are a few vicilin structures determined from jackbean, french bean, soybean and peanut, respectively^18^,^19^,^20^,^21^. It is apparent that there could be many more physiological functions of vicilins that are yet to be identified. Comparative structural proteomics studies will help identify other possible physiological roles of vicilins or vicilin-like proteins. The structural insights themselves can prove to be equally interesting, as we found in the case of SM80.1 from *Solanum melongena* (eggplant) seeds.

The vicilin, SM80.1, from *S. melongena* was purified and the crystal structure determined using *ab initio* phasing method. Crystal structure showed presence of acetate and pyroglutamate molecules bound in structurally quasi-equivalent cavities in each of the two domains of SM80.1. To the best of our knowledge, the anionic ligands bound to 7S vicilin have been identified for the first time. Association of such specific moieties indicates possible roles of this protein in critical metabolic pathways, in addition to helping protein’s architectural stability. We were able to visualize a highly flexible loop region, which is otherwise missing or found disordered in other homologous vicilin structures. Indeed, vicilin-like fold exists in a wide range of organisms and is involved in variety of different functions.

## Results and Discussion

Comparative structural biology of seed proteins has not been adequately explored, in spite of these being most abundant and easily accessible. Therefore, the available structure-function data of seed proteins are limited. Systematic studies involving purification of individual proteins and comparative structural analyses would provide greater physiological insights concerning their functions^15^. *S. melongena* (eggplant) is among the most abundantly used plant fruits which are rich in seeds. The protein composition of seeds of this plant, therefore contributes significantly to human health. Systematic analysis of the seed proteome was therefore initiated.

### Protein purification and crystallization

Whole proteome of *S. melongena* seeds was subjected to fractionation followed by purification and crystallization of a dominant protein SM80.1. Washed, delipidified and grounded *S. melongena* seeds were subjected to ammonium sulphate fractionation over the range of 0 to 95%, which resulted into selective precipitation of different proteins on the basis of solubility. All fractions were electrophoresed along with low molecular weight markers (Sigma) (Supplementary Figure S1a). Five major protein bands were selected and transferred on PVDF membrane for characterization. Transferred proteins were stained with Ponceau S and subjected to Edman degradation for identification of N-terminal amino acid sequence. Band 1 showed significant homology with an allergy-related protein from *Solanum lycopersicum*^22^. Bands 2 and 3 showed homology with hypothetical protein from *Vitis vinifera* whereas bands 4 and 5 belonged to 11S globulin family and have similarity with 11S legumin from *Sesamum indicum* (Table 1). Based on the limited N-terminal sequence and abundance, band 1 was selected for further purification and characterization which was discussed in our previous paper^23^. Purification profile of 45 kDa band 1 SM80.1 protein by size exclusion chromatography is shown in Supplementary Figure S1b.

**Figure 1:**
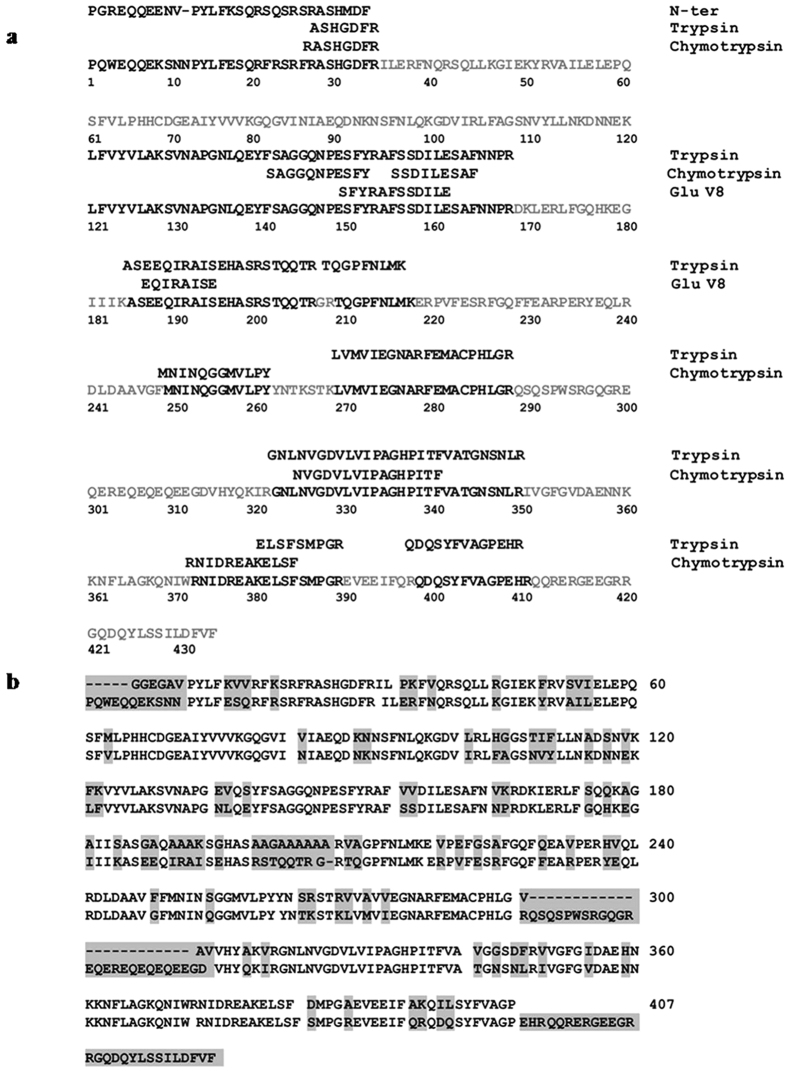
Sequence alignment of SM80.1 protein. (**a**) The sequence alignments of peptides obtained by MASCOT database search utilizing MS-MS of proteolytically (Trypsin, Chymotrypsin, GluV8) digested protein fragments with protein sequence of 7S vicilin from *Solanum lycopersicum*. N-terminal is obtained by Edman degradation chemistry. Residues that are non-identified between the two proteins are marked in grey. (**b**) The alignment of amino acid sequence of SM80.1 identified on the basis of crystal structure with sequence obtained from mass spectrometry by MASCOT database search and N-terminal. Different residues among the sequences are highlighted with grey background.

To acquire the full length sequence of SM80.1, proteolytic digestion was carried out. Enzymes like trypsin, chymotrypsin, endoproteinase Glu V8 were used for protein digestion to generate short peptide fragments (Fig. 1a). These fragments were vacuum-dried and then passed through nano LC-mass spectrometer followed by data processing. Sequence alignment obtained from search engine MASCOT revealed that this protein has about 85% sequence homology with the vicilins of *S. lycopersicum*.

Further, crystallization of the purified protein was attempted as reported earlier^23^. Another crystal form was also obtained which diffracted to a higher resolution. Two different crystal forms of SM80.1 thus obtained, belonged to P6_3_22 and R32 space groups with the unit cell dimensions a = 117.9 Å, c = 123.5 Å and a = 119.4 Å, c = 158.0 Å, respectively. All subsequent crystallographic analyses were carried out using only the R32 crystal form that diffracted at higher resolution. The data collection and processing statistics are summarized in Table 2. The asymmetric unit of SM80.1 crystals contains one monomer in the asymmetric unit with 49.0% solvent content, as calculated on the basis of Matthews coefficient.

### Structure determination

We tried *ab initio* S-SAD phasing method for determining the preliminary phases. Eight inherent sulphur atoms (six methionines and two cysteines) were used. PHENIX package^24^ was utilized to extract the initial phase information from experimental anomalous intensities. For the purpose of SAD phasing, three different kappa 0, 40, 80, and fine-slicing oscillations were taken into consideration which contributed to 64.8 fold redundancy (Table 2). A preliminary partial poly-Ala model was built automatically by PHENIX Autosol software and it was further enhanced using AutoBuild through iterative model building and refinement. A total of 225 residues were built and 30 chains were placed with *R*_work_ and *R*_free_ values of 46.0% and 50.0% respectively. Model completion, addition of waters and occupancy refinement were carried out using COOT^25^ and CNS^26^. The model was refined to *R*_work_ of 19.94% and *R*_free_ of 21.02% with 98.5% of the residues in the allowed region of the Ramachandran plot. Remaining residues are labeled as outlier and are present in loop region. This region shows high flexibility with relatively poor electron density. The final refinement parameters are listed in Table 3. Protein sequencing methods and interpretation of electron density map at a resolution of 1.5 Å identified the complete sequence of SM80.1 (Fig. 1b).

### Overall Structure

**Figure 2:**
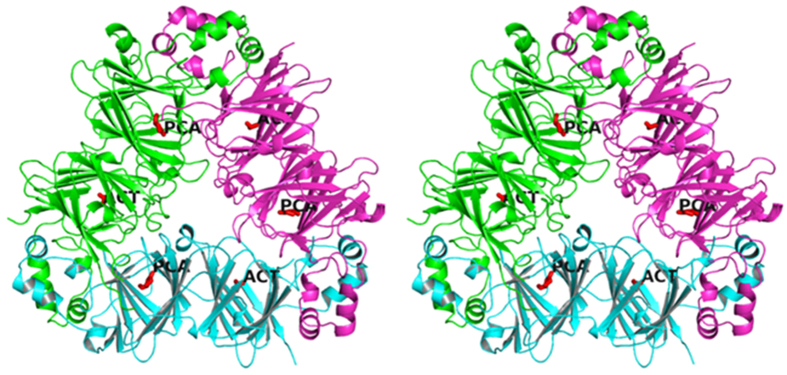
Stereo view of ribbon diagram of the trimeric protein along three fold axis of symmetry showing the overall structure of SM80.1 protein. PCA and ACT are pyroglutamate and acetate identified in two domains of SM80.1 protein.

The overall crystal structure of SM80.1 consists of 392 residues of which the region 287–297 is structurally disordered and showed broken density whereas the core is clearly resolved. SM80.1 structure consists of α-helices, β-sheets and flexible loops. There is one monomer in the asymmetric unit. Further, examination of the region around the asymmetric unit in crystal structure revealed that the protein exists as a homotrimer. This trimer formation around the threefold crystallographic axis, as shown in ribbon diagram (Fig. 2), resembles that in the other vicilins. The trimerization helps in providing thermostability to the protein^11^. Each monomer also shows formation of core region by pair of β-barrels and helices protruding outward from each core to interact with the neighboring monomers, resulting in the formation of a trimer. It is observed that quaternary structure is stabilized by electrostatic interactions.

Monomer can further be divide across a pseudo-dyad axis into two similar halves, having a core region and extended arms, forming two domains. These two domains are named N- and C-terminal domains. Core region of each domain is formed by β-strands whereas the helices are involved in the formation of extended arms. Apart from these secondary structures, short β-turn is also present in the N-terminal of SM80.1 protein structure. Magnesium (Mg) ion is also present in the structure and exists at the interface of the monomers. The chemical environment of Mg ion is favoured by Gly28 and Ala25 with 3 water molecules present at a distance of 2.36, 2.34, 2.44, 2.43 and 2.39 Å, respectively. B-factor of the magnesium ion is 14.77 Å^2^ which is low in comparison to overall B-factor of protein or water molecules. This is because coordinating residues also show low B-factors (B_avg_ = 14.31 Å^2^). Anomalous difference Fourier map was calculated using “SAD” data to check the position of Mg ion and Sulphur atoms. The anomalous difference Fourier map identified peak height of 4.5 σ for magnesium ion. Also, all the sulphur atoms were identified at correct positions with an average peak height of 16.63 σ. This information is reflected in the supplementary material (Supplementary Table S1). The final model has 256 water molecules. More than 95% residues, i.e. around 359 out of 384 residues are surface exposed with overall surface area being around 17750.3 Å^2^.

Apart from electrostatic interactions, hydrophobic interactions at each monomer-monomer interface are critical in stabilization of the tertiary structure of SM80.1. Hydrophobic residues form hydrophobic patches which are involved in trimer formation by locking the extended arms. This suggested importance of these interactions for the formation of quaternary structure in this family of proteins.

### Structural comparisons with related proteins

**Figure 3:**
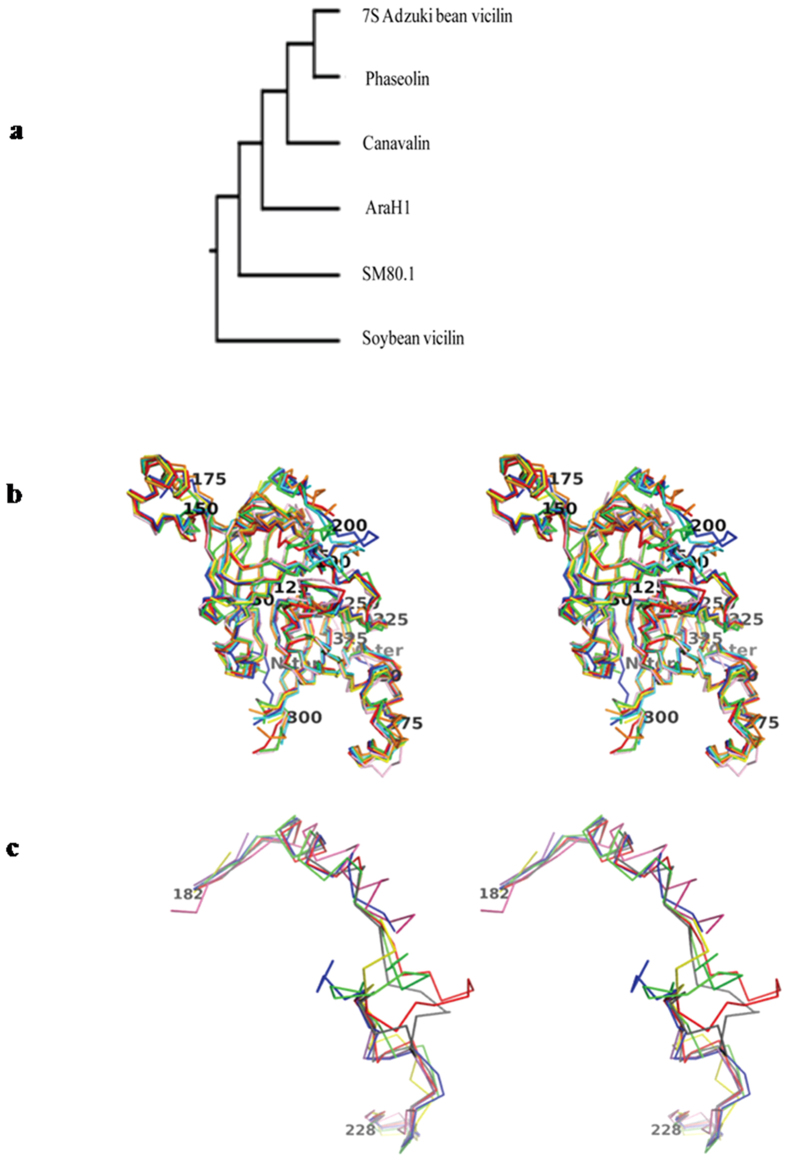
Cα comparison of structures homologous to SM80.1 protein. (**a**) Dendrogram of high fold similarity structure of SM80.1 with 7S vicilin of Adzuki bean, Phaseolin, Canavalin, AraH1 and Soybean. (**b**) Monomer superimposition of Cα chain of SM80.1 (green), Adzuki bean (blue), AraH1 (red), Phaseolin (orange), Canavalin (yellow), Soybean (cyan) and Korean pine vicilin (pink). N-terminal, C-terminal and every consecutive 25^th^ amino acid of SM80.1 vicilin is labeled. (**c**) Superimposition of comparative loop of all the above structures.

Dali server (DaliLite ver. 3) was used for identifying structural correlation with other canonical proteins. Protein structure based search engine^27^, indicated many structures similar to SM80.1 with very low sequence homology. Supplementary Table S2 shows that these proteins have below 30% sequence homology indicating that a common structural fold is being used, perhaps for different functions. A phylogenetic tree of all these proteins explains the relationship and diversification among the members (Fig. 3a). Structural comparisons showed that vicilins AraH1 (pdb: 3SMH), 7S globulin-1 adzuki bean (pdb: 2EA7), β-conglycinin (pdb: 1UIJ), 8S mungbean storage protein (pdb: 2CV6), Korean pine vicilin (pdb: 4LEJ), canavalin (pdb: 2CAV), phaseolin (pdb: PHL) have high structural homology (RMSD less than 2 Å) with SM80.1^18^,^20^,^21^,^28^,^29^,^30^,^31^. Dali analysis also suggested that similar fold is spread across in all the species. It is evident that *Bacillus subtilis* (pdb: 1L3J), *Pseudomonas aeruginosa* (pdb: 1SQ4), *Geobacillus kaustophilus* (pdb: 2P17), *Marinobacter aquaeolei* (pdb: 3O14), *Xanthomonas campestris* (pdb: 3H50), *Chlamydomonas reinhardtii* (pdb: 2V4A) as well as *Homo sapiens* (pdb: 2W2I) exhibit similar fold although with weak homology. To have better understanding, carbon alpha (Cα) chains of monomeric unit of these proteins were superimposed (Fig. 3b), leading to identification of flexible loop with high B-factors. Superimposition of this particular loop is shown in Fig. 3c. High structural homology of SM80.1 with many other vicilins did not significantly reflect in sequence. AraH1 from peanut and 7S adzuki bean vicilin showed highest structural similarity with SM80.1 with RMSD value of 1.4 Å and 1.3 Å, respectively as analysed by Dali server for complete trimeric molecule.

As in the case of most vicilins, SM80.1 also showed presence of hydrophobic interactions playing critical role in trimer formation and stability, thus protecting epitopes from digestive enzymes^21^. Comparison of SM80.1 with adzuki bean 7S vicilin^28^ monomer using PyMol shows RMSD of 0.979 Å (Fig. 3b). SM80.1 shows difference in position of short β-turns which are present at the N-terminus and before first β-strand whereas in adzuki bean 7S vicilin a short β-turn is present after first β-strand. In the case of adzuki bean 7S vicilin, loops corresponding to residues Glu188 to Glu196 and Ser222 to Ser227 are disordered, which is not the case with SM80.1, although the corresponding residues show comparatively higher B-factors (Supplementary Figure S2).

### Metal binding

**Figure 4:**
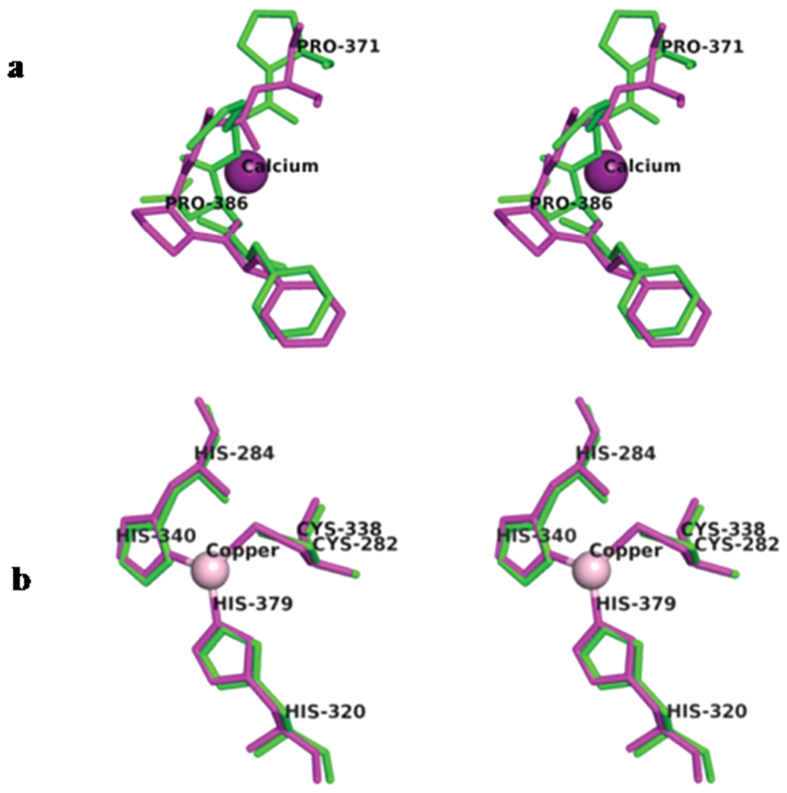
Stereo view comparison of the metal binding site in SM80.1 with other homologous vicilin. (**a**) Helix loop helix motif of SM80.1 (green) and adzuki bean 7S vicilin (magenta). (**b**) Copper binding site in SM80.1 (green) and Korean pine vicilin (magenta).

SM80.1 lacks bound calcium despite its structure being similar to that of 7S adzuki bean vicilin, a protein showing bound calcium^28^. This is probably due to a substantial difference in the geometrical arrangement of side chains at the canonical calcium binding site. Thus, even slight variation in this local sequence can disallow calcium binding, although binding motif of helix-loop-helix is conserved. Indeed, this topological arrangement of side chains is important for metal binding (Fig. 4a). On the contrary, sometimes even after conservation of geometrical arrangement of side chains, metal binding is not observed. For example, bound copper is not found in SM80.1 although residues required for coordinating copper are present. On the other hand, similar residues i.e two histidines and one cysteine are favouring existence of copper center in Korean pine vicilin (Fig. 4b)^30^. Careful analysis in SM80.1 structure around the positions where calcium or copper were present in other homologous proteins, 7S adzuki bean vicilin and Korean pine vicilin, indicating absence of electron density in difference maps implying absence of bound calcium or copper, in SM80.1. Above analyses indicate subtle but critical variations in 7S vicilins, a possible indication that it is associated with a variety of functions, which include assisting metabolites and other related molecules during germination and growth.

### Bound metabolites

**Figure 5:**
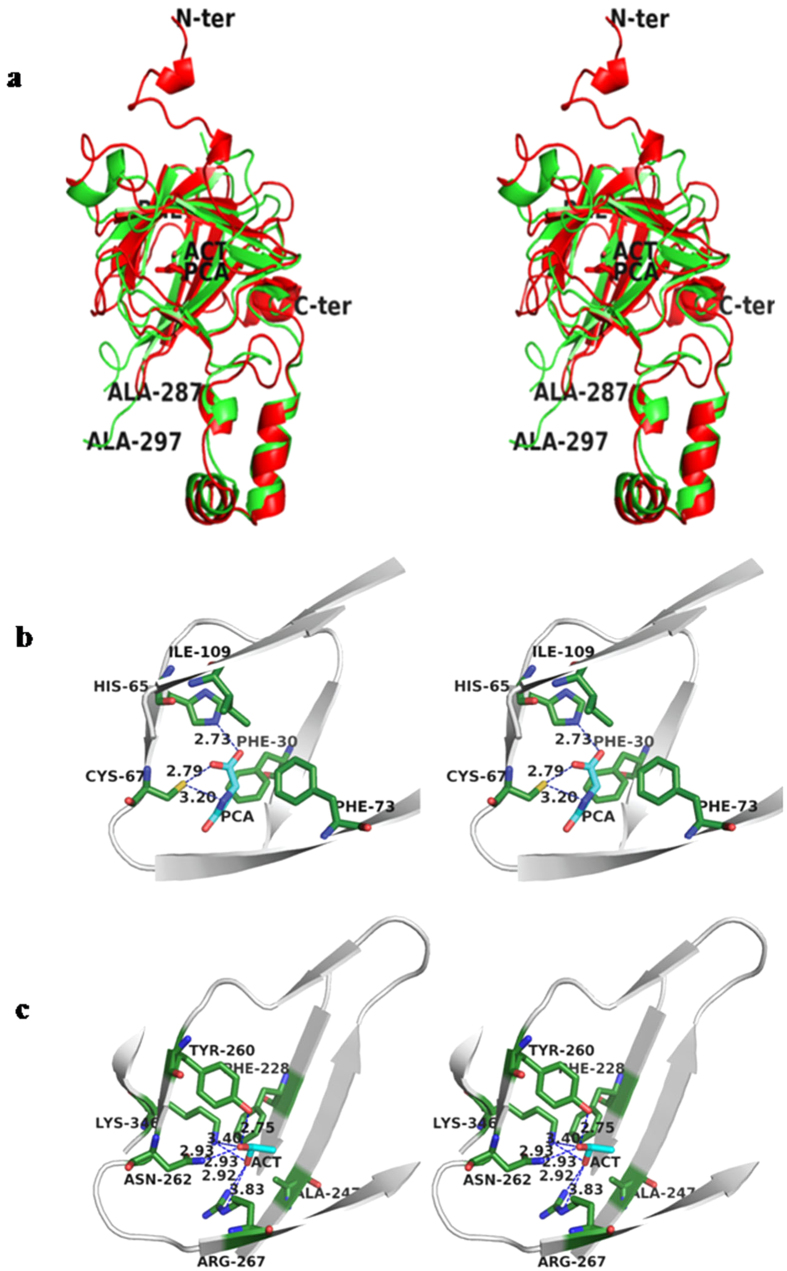
Stereo view of anionic ligand binding pockets in SM80.1. (**a**) Superimposition of binding pocket of two domains showing the plasticity for different ligands. (**b**) Pyroglutamate binding pocket with pyroglutamate (PCA) present in center surrounded by interacting residues. (**c**) Acetate binding pocket with acetate present in the central cavity and interacting residues are labeled in black.

We were able to identify the bound ligands based on the high resolution electron density map at 1.5 Å resolution. Both the N- and the C-terminal domains of SM80.1 form a central cavity each. In these predominantly hydrophobic and structurally similar cavities, two different ligands were observed (Fig. 5a). The N-terminal domain has cyclic lactam of glutamate, called pyroglutamate. Acetate moiety was present in the corresponding pocket in the C-terminal domain (Supplementary Figure S3). It appears that the two ligands may be contributing to the structural stability of the protein. Presence of metabolite in the cavity is consistent with metabolic profiling data that identified pyroglutamate in the extract of tomato fruit and seeds^32^.

Pyroglutamate is present in the largely hydrophobic core of N-terminal β-barrel domain (Fig. 5b). Surface area occupied by pyroglutamate is around 249.8 Å^2^. Pyroglutamate is stabilized by three electrostatic interactions. Pyroglutamate (N), (O) and (OXT) are forming electrostatic bond with Cys67, His65, Cys67 at a distance of 3.20 Å, 2.73 Å, 2.79 Å (Supplementary Table S3). With buried surface area of 243.92 Å^2^ out of the total accessible surface area (ASA) of 249.77 Å^2^, pyroglutamate is completely buried. Phe30, His65, Cys67, Tyr73 and Ile109 are the residues in the close proximity and interacting with pyroglutamate in the N-terminal core region.

In the core cavity of the C-terminal β-barrel, clearly evident was the presence of an acetate moiety fully buried, occupying around 187.1 Å^2^ surface area (Fig. 5c). Acetate is making in total six electrostatic interactions, three each with [O] and [OXT]. Acetate [OXT] is forming bond with Asn262, Tyr260 and Lys346 at a distance of 2.93 Å, 2.75 Å and 3.40 Å respectively. Similarly, acetate [O] is forming bond with Lys 346 and two bonds with Arg267 at a distance of 2.93 Å, 2.92 Å and 3.83 Å respectively. Detailed statistics of ligand electrostatic interaction are enumerated in Supplementary Table S3. Superimposition of the ligand binding sites in the two domains is shown in Fig. 5a. It is evident that both the ligands exist at equivalent positions in the largely hydrophobic pockets and are stabilized by electrostatic interactions involving charged residues.

On comparison of bound ligand with other vicilins, it is observed that glycerol is found in Korean pine vicilin at the position where pyroglutamate is present in SM80.1. However, this pocket is empty in AraH1, Canavalin, Phaseolin and in β-conglycinin. But, when the electron density was evaluated in the corresponding pocket in the molecule of adzuki bean 7S vicilin, an unexplained density was seen around the same position, suggesting that it was not analysed in the light of such a molecule. Therefore, possibility of presence of pyroglutamate cannot be avoided in adzuki bean 7S vicilin on the basis on unexplained density. Thus, both SM80.1 and probably adzuki bean 7S vicilin have pyroglutamate or similar metabolite in the N-terminal domain and acetate molecule within same region in the C-terminal β-barrel domain.

### Physiological implication

Structural analysis identified putative binding pocket for anionic ligands in the predominantly hydrophobic core. In addition to playing a role in maintaining structural integrity of the protein and probably serving as energy source, they are likely to be involved in metabolic pathways in temporal fashion.

Pyroglutamate, an intermediate metabolite has not been discovered earlier in any other vicilin structure. As such, pyroglutamate is ubiquitously present in living cells ranging from archaebacteria to humans and is involved in all glutamate linked processes^33^. Apart from N-terminal modification, pyroglutamate also exists as free cellular metabolite. Free cellular pyroglutamate may act as an analogue or precursor of glutamate. On the other hand, acetate the other metabolite present in SM80.1 probably meets the requirement of oil as precursor moiety^34^,^35^.

In SM80.1, pyroglutamate and acetate could have role in Calvin cycle and further, pyroglutamate may be involved in glutathione metabolism. Calvin cycle is one of the cardinal metabolic pathways in plants^36^. Acetate is required in Calvin cycle and is produced by protein catabolism^37^. Oxidation of acetate leads to the formation of central metabolite of Calvin cycle i.e. acetyl-CoA^38^. This undergoes series of chemical reactions and forms an intermediate product 2–oxoglutarate/α-ketoglutarate which acts as a precursor for the biosynthesis of pyroglutamate^39^. Pyroglutamate could be further involved in glutathione metabolism and play a critical role in oxidative stress^40^.

The fact that these two closely linked metabolites are found in bound form with SM80.1 suggests attractive possibility of this protein being important in stress management. Also due to the inert nature and high water binding capacity, pyroglutamate is an excellent candidate to serve as an osmoprotectant^33^, a property appropriate for stress management. Acetate and pyroglutamate both act as transitional partners and play a role in metabolic pathways thereby suggesting that they assist in growth and germination of seeds.

## Materials and Methods

### Protein purification

Eggplant *(Solanum melongena*) seeds were procured from the National Seeds Corporation, Indian Agricultural Research Institute (IARI), New Delhi, India. Seeds (50 g) were grounded to fine powder and defatted using petroleum ether. Defatted powder was then homogenized with 50 mM Tris-HCl, pH 8 containing 150 mM NaCl by continuous stirring for 4 hrs at 277 K in the presence of a protease inhibitor cocktail (Sigma). The crude extract was prepared by centrifugation at 7000 rpm for 3 hrs and then subjected to salt fractionation over a concentration range of 0–95% (w/v) ammonium sulphate. Pellets thus obtained at each step were resuspended in 50 mM Tris-HCl buffer pH 8 and dialyzed to remove the traces of salt. Resuspended pellets were further electrophoresed on 15% SDS PAGE to identify the proteins present in each fraction. The isolation and purification of proteins was carried out from 80% ammonium sulphate fraction by gel-filtration chromatographic technique, using manually packed sephacryl-200 column, pre-equilibrated with 50 mM Tris-HCl, pH 8. Protein concentration at different levels of purification was determined by BCA protein estimation assay.

### Protein sequencing

To identify the complete sequence of the purified protein by internal sequencing trifluoroethanol (TFE) protein digestion protocol was followed with slight modification (Agilent Technologies). 250 μg of protein was dissolved in 100 mM ammonium bicarbonate buffer and denatured by TFE. It was then reduced and denatured at 333 K by addition of 200 mM DTT for 45 min. Subsequently, alkylation was done in the dark by addition of 200 mM iodoacetamide for an hour at room temperature. DTT was again added to destroy the excess of iodoacetamide in dark for one hour. Further, milliQ water was added to dilute the denaturant, and ammonium bicarbonate solution was used to raise the pH. Different enzymes were used to digest the protein at 1:50 enzyme:substrate concentration and all solutions were incubated overnight at 298 K. Absolute formic acid was used to lower the pH and stop the enzyme activity. The resulting solutions were dried in a vacuum centrifuge and resuspended in 50% acetonitrile +0.1% formic acid. The digest was diluted and analyzed by mass spectrometry.

### Mass Spectrometry

Samples were subjected to an Eksigent Nano LC ultra 2D plus system (AB Sciex, Waldbronn, Germany) connected to Hybrid Quadrupole-TOF LC/MS/MS Mass Spectrometer (AB Sciex, Waldbronn, Germany). The calibration of system was done using beta-galactosidase and combined with C18 trap column and C18 RP analytical column for the analysis of digested peptide fragments. Sample trapping and washing was carried out at a flow of 5 μl in 12 min run time with 100% solvent A (water +0.1% formic acid), and elution was accomplished during a 45 min. gradient from 13% to 32% Solvent B (ACN +0.1% formic acid) at 550 nL/min. The positive ion mode was selected for running the sample, with MS window from 350 to 1250 *m/z*. Parent ions were fragmented by Atmospheric Pressure Ionization. The flow rate was split less and the column flow through was directly introduced into the Nano spray III ion source. MS-MS data were extracted for database searches.

### Database searches

MS-MS data analysis and generation of peak list were performed with the protein pilot software (version 4.5, Applied Biosystems). Highly specific rigorous searches (MASCOT) were useful to identify either known proteins (*i.e.* in database), or unknown proteins (identical peptides). For the analysis, peptide precursor tolerance was set to 100 ppm for 2 and 3 peptide charges (ESI), and MS/MS tolerance was 0.9 Da (ESI) within MASCOT parameters. Also carbamidomethyl (C) was marked as fixed modification whereas oxidation (M) and deamidation (NQ) as variable modifications. Sequence alignment was carried out using Clustal W^41^. Sequence of vicilin from *Solanum lycopersicum* was used as a guide to align the sequences of proteolytic fragments obtained from MASCOT. The part of sequence which was not sequenced biochemically was identified crystallographically using high resolution electron density map.

### Crystallization

Initial crystallization trials for the purified protein (10 mg/ml) involved the exploration of various precipitants, detergents, concentrations and pH values. The final diffraction data were collected from crystals grown in a drop composed of 2 μl protein solution with 2 μl reservoir solution containing 10% (w/v) PEG 3350, 1.5 M–2 M Sodium malonate by hanging drop vapour diffusion at 25°C. Surprisingly, two different crystal forms were obtained in above conditions. Since Crystallization condition contains sodium malonate, no cryoprotectant was used for data collection.

### Data Collection and processing

For *ab initio* structure solution, inherent sulphur atoms present in the protein were utilized for single anomalous diffraction (SAD) phasing^42^. Data for both native and SAD were collected at beamline BM14, European Synchrotron Radiation Facility (ESRF), Grenoble, France at wavelengths of 0.953 Å and 1.771 Å respectively. The data were collected at 120 K with crystal-to-detector distance of 120 mm, an oscillation range of 0.5° and an exposure time of 2 sec per image. Data were integrated and scaled with *imosflm* or HKL2000. Rmeas, Rpim and CC1/2 were extracted by using merging statistics tool of PHENIX software suite (Phenix version 3.0.1).

### *Ab initio* structure solution

Although molecular replacement could be done using the protein structures having weak homology with 7S vicilins as discussed in our earlier paper^23^, we exploited the initial phase information by S-SAD phasing method to solve the structure. Two data sets at 1.5 Å and 2.18 Å resolution were collected from the same crystal, at 120 K. To increase the accuracy, high multiplicity of the data at the 2.18 Å resolution (64.8-fold) and fine slicing rotations (0.5°) and three different kappa angles were used. Single-wavelength anomalous diffraction (SAD) using sulphur was carried out for the determination of phases and structure was refined. Data were indexed, integrated and final scaling for the native data was carried out by SCALEPACK. For merging, SAD datasets were also processed with same settings as the native data set. The PHENIX software suite^24^ module, AutoSol was used to locate the anomalous scatterers from the S-SAD intensity data, calculating the initial experimental phases, density modification and preliminary model building. High resolution data were used by AutoBuild for improving the phases and model building. COOT^25^ was used to build the missing residues and side chains of the model. CNS^26^ was used for B-factor and minimization. The theoretical Bijvoet pairs were calculated using the modified Hendrickson formula in Autosol program of PHENIX software^42^,^43^. The quality of atomic model was assessed with PROCHECK^44^. The figures were generated using PyMOL^45^. The final model and structure factors have been deposited in protein data bank (PDB ID: 5CAD).

## Additional Information

**How to cite this article**: Jain, A. *et al.* Crystal structure of the vicilin from *Solanum melongena* reveals existence of different anionic ligands in structurally similar pockets. *Sci. Rep.* **6**, 23600; doi: 10.1038/srep23600 (2016).

## Supplementary Material

Supplementary Information

## Figures and Tables

**Table 1:** N-terminal sequence of different protein bands.

Segregated Proteins	N-terminal Sequence	Homologous Proteins
Band 1	PGREQQEENVPYLFKSQRSQSRSRASHMDF	Allergenic vicilin from *Solanum lycopersicum*
Band 2	YKEYPGQHGQQGQTGI-P/I-LTXQARHQR/V	Hypothetical protein sorbidraft, *Vitis vinifera*
Band 3	GLEENIQTTKIRTNMEEYYYADIYVI	Hypothetical protein *Vitis vinifera*, *Arabidopsis lyrata*
Band 4	GLEETIRSAKLRENNDNPPAAADVYNPQGG	11S globulin storage *Sesamum indicum*
Band 5	GIEETYTMKLRENIGHPXXXDDVNNPRGR	11S globulin storage *Sesamum indicum*

**Table 2:** Data collection and S-SAD phasing statistics.

	Dataset1 SAD 0 Kappa*	Dataset2 SAD 40 Kappa*	Dataset3 SAD 80 Kappa*	Merged SAD*	Dataset4 Native#
Space group	*R32*	*R32*	*R32*	*R32*	*R32*
Unit-cell parameters					
* a* (Å)	119.49	119.47	119.45	119.48	119.36
* b* (Å)	119.49	119.47	119.45	119.48	119.36
* c* (Å)	158.09	157.95	157.93	158	158
Wavelength (Å)	1.771	1.771	1.771	1.771	0.95372
*Detector distance*	102.17	102.17	102.17	–	119.30
*Oscillation*	1	1	1	–	0.5
No of images	360	360	360	1080	400
Total reflections	494720 (45039)	492603 (45736)	490806 (42860)	1479455 (134513)	829874 (106878)
Unique reflections	22886 (2237)	22714 (2212)	22847 (2230)	22848 (2242)	70495 (10235)
Resolution range (Å)	50–2.18	50–2.18	50–2.18	50–2.18	22.71–1.49
Completeness (%)	100.0 (99.6)	99.8 (98.4)	99.9 (99.4)	100 (99.1)	99.9 (100)
Redundancy	21.6 (20.1)	21.7 (20.7)	21.5 (19.2)	64.8 (60.0)	11.8 (10.4)
Mean *I*/(*I*)	32.9 (7.8)	36.0 (7.2)	34.3 (6.2)	59.8 (12.3)	21.1 (4.3)
*R*_*merge*_† (%)	8.5 (40.1)	8.8 (44.9)	9.1 (46.9)	8.8 (44.7)	6.3 (49.9)
*R*_*meas*_††	8.7 (41.1)	9.0 (46.0)	9.3 (48.1)	8.9 (45.1)	6.5 (52.4)
*R*_*pim*_†††	1.9 (9.1)	1.9 (10.1)	2.0 (10.9)	1.1 (5.8)	1.9 (15.9)
* CC1/2*	0.999 (0.984)	0.999 (0.984)	0.999 (0.978)	1.000 (0.994)	0.999 (0.934)

^†^R_merge_ = Σ_hkl_Σ_i_|I_i_(hkl) − <I(hkl)> |/Σ_hkl_ Σ_i_I_i_(hkl) where I_i_(hkl)is the i^th^ measurement of the intensity of reflection hkl and <I(hkl)> is the mean intensity of reflection hkl.

^††^R_meas_ (redundancy-independent R_merge_) = Σ_hkl_[N_hkl_/(N_hkl_ − 1)]^1/2^Σ_i_|I_i_(hkl) − <I(hkl)> |/Σ_hkl_ Σ_i_I_i_(hkl).

^†††^R_pim_ (precision-indicating R_merge_) = Σ_hkl_[1/(N_hkl_ − 1)]^1/2^Σ_i_|I_i_(hkl) − <I(hkl)> |/Σ_hkl_ Σ_i_I_i_(hkl).

^*^Data processed using HKl2000 and merging statistics calculated using Phenix.

^#^Data processed using *imosflm.*

**Table 3:** Refinement statistics.

Resolution range (Å)	22.71 −1.49
No. of reflections, working set	66896
No. of reflections, test set	3556
Final *R*_work_ (%)	19.94
Final *R*_free_ (%)	21.02
No. of non-H atoms	
Protein	2912
Ion	1
Ligand	
ACETATE	4
PYROGLUTAMATE	9
Water	256
Total	3182
R.M.S. deviations	
Bonds (Å)	0.009
Angles (°)	1.97
Average *B* factors (Å^2^)	
Protein	20.51
Ion	14.770
Ligand	
ACETATE	14.343
PYROGLUTAMATE	46.509
Water	32.08
Ramachandran plot	
Most favoured (%)	92.1
Allowed (%)	6.4
Outlier (%)	1.5
