# Supplementary material for: Crystal structure of the vicilin from Solanum melongena reveals existence of different anionic ligands in structurally similar pockets
Source: Sci Rep. 2016 Mar 23;6:23600. doi: 10.1038/srep23600 (PMC4804240; doi:10.1038/srep23600)
Supplement: Supplementary Information [file srep23600-s1.doc]

**Crystal structure of the vicilin from *Solanum melongena* reveals existence of different anionic ligands in structurally similar pockets**

Abha Jain^1,2^, Ashish Kumar^1,3^, Dinakar M. Salunke^1,4*^

^1^ Regional Centre for Biotechnology, Faridabad-121001 India

^2^Manipal University, Manipal, Karnataka-576104, India

^3^National Institute of Immunology, New Delhi-110067, India

^4^International Centre for Genetic Engineering and Biotechnology, New Delhi-110067, India

^*^Corresponding author: Dinakar M. Salunke

International Centre for Genetic Engineering and Biotechnology,

New Delhi-110067

Telephone number: +91 11 26742317

Fax number: +91 11 26742316

E-mail address: dmsalunke@rcb.res.in

**Supplementary Figures**

**
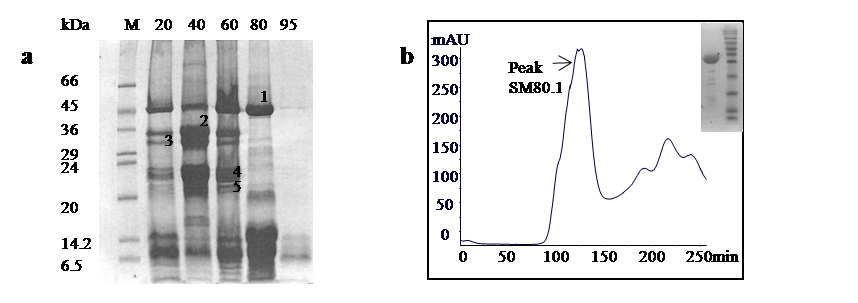
**

**Supplementary Figure 1**: Purification and characterization of SM80.1 protein **a)** SDS-PAGE showing the composition of the fractions obtained by ammonium sulphate fractionation of *S. melongena* seed aqueous extract. **b)** Purification of 45 kDa SM80.1 protein by size exclusion chromatography on manually packed Sephacryl-200 column. The peak-1 corresponds to the SM80.1 protein of *S. melongena* purified from 80 % ammonium sulfate cut and in inset is the reducing SDS-PAGE of the purified protein. M: molecular markers

**
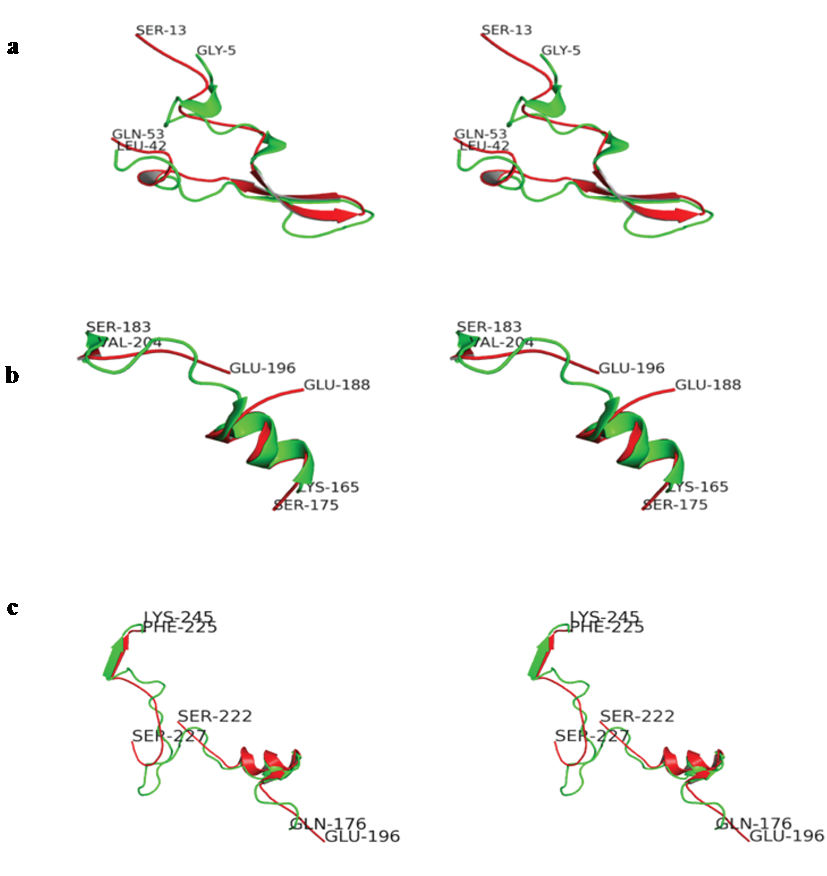
**

**Supplementary Figure 2:** Comparative analysis of SM80.1 (Green) and 7S Adzuki bean vicilin (Red). **a)** N-terminal superimposition highlighting the loop difference in two vicilin. **b)** SM80.1 showing complete loop in region 165-183. In 7S Adzuki bean vicilin structure this loop, residue Glu188 to Glu196 is missing. **c)** Visualization of a highly flexible loop region in SM80.1 which is missing in 7S adzuki bean vicilin structure and many other vicilins.

**
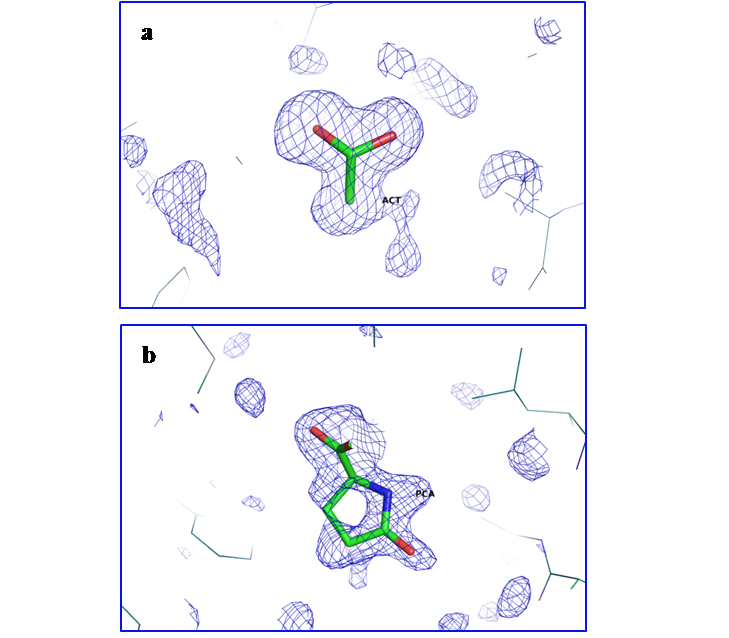
**

**Supplementary Figure 3:** Ligand electron density in SM80.1**. a)** Ligand omit Fo-Fc electron density map of pyroglutamate at 1.8 σ level. **b)** Ligand omit Fo-Fc electron density map of acetate at 1.8 σ level. Figure is prepared in pymol

**Supplementary table 1: Statistics of peak height of the anomalous map at the ion position as well as sulphur atom calculated using SAD Anomalous difference Fourier map.**

| **S. No** | **Residue** | **Peak height (sigma)** |
| --- | --- | --- |
| 1 | Met62 | 22 |
| 2 | Cys67 | 12 |
| 3 | Met215 | 8.50 |
| 4 | Met249 | 21 |
| 5 | Met256 | 22.7 |
| 6 | Met280 | 18 |
| 7 | Cys282 | 15.30 |
| 8 | Met370 | 13.50 |
| 9 | Magnesium Ion | 4.5 |

**Supplementary table 2: Sequence comparison with other homologous proteins**

| Similar Protein | % identity | RMSD (Dali Server) |
| --- | --- | --- |
| 7S Adzuki bean vicilin | 26.2 | 1.3 |
| Phaseolin | 19.2 | 1.7 |
| AraH1 | 26.2 | 1.4 |
| Soybean vicilin | 13.2 | 1.5 |
| Canavalin | 26.2 | 1.3 |

**Supplementary table 3: Ligand electrostatic interactions statistics**

|  | | **Residue** | | **Atom** | | **Distance (Å)** | |  |
| --- | --- | --- | --- | --- | --- | --- | --- | --- |
| **PYROGLUTAMATE** | |  | |  | |  | |  |
| 1[OXT] | | Cys67 | | SG | | 2.79 | |  |
| 1[O] | | His65 | | NE2 | | 2.73 | |  |
| 1[N] | | Cys67 | | SG | | 3.20 | |  |
| **ACETATE** | | |  | |  | |  | |
| 1[OXT] | Asn262 | | | | ND2 | | 2.93 | |
| 1[OXT] | Tyr260 | | | | OH | | 2.75 | |
| 1[OXT] | Lys346 | | | | NZ | | 3.40 | |
| 1[O] | Lys346 | | | | NZ | | 2.93 | |
| 1[O] | Arg267 | | | | NH1 | | 2.92 | |
| 1[O] | Arg267 | | | | NE | | 3.83 | |
